# Associations between velamentous or marginal cord insertion and risk of adverse perinatal outcomes in twin pregnancies: a retrospective cohort study

**DOI:** 10.1186/s12884-023-05963-1

**Published:** 2023-09-08

**Authors:** Li Wen, Qimei Zhong, Lingwei Mei, Li Gao, Xia Lan, Jing Xiong, Shujuan Luo, Lan Wang

**Affiliations:** 1 Department of Obstetrics and Gynecology, Women and Children’s Hospital of Chongqing Medical University, Chongqing, 401147 China; 2 Department of Obstetrics and Gynecology, Chongqing Health Center for Women and Children, Chongqing, 401147 China

**Keywords:** Velamentous cord insertion, Marginal cord insertion, Selective fetal growth restriction, Small-for-gestational age, Preeclampsia, Twin pregnancies

## Abstract

**Background:**

Velamentous cord insertion (VCI) and marginal cord insertion (MCI) are well-known risk factors for adverse perinatal outcomes in singleton pregnancies. However, the potential links between VCI or MCI and perinatal outcomes in twin pregnancies have yet to be systematically evaluated. This study aimed to investigate the relationships between VCI or MCI and perinatal outcomes in twin pregnancies.

**Methods:**

This retrospective single-center cohort study included women with twin pregnancies who gave birth in a tertiary hospital in Southwest, China between January 2017 and December 2022. VCI and MCI were identified by abdominal ultrasound and confirmed after placental delivery. Logistic regression, multinomial logit regression and generalized estimation equation models were used to evaluate the association between VCI or MCI and perinatal outcomes.

**Results:**

A total of 3682 twin pregnancies were included, including 100 (2.7%) pregnancies with VCI and 149 (4.0%) pregnancies with MCI. Compared to pregnancies with normal cord insertion, both monochorionic and dichorionic pregnancies with VCI were associated with an increased risk of preterm delivery 32–34 weeks (aRRR 2.94, 95% CI 1.03–8.39; aRRR 2.55, 95% CI 1.19–5.46, respectively), while pregnancies with MCI were not associated with preterm delivery. VCI was associated with a higher incidence of placental previa (aOR 6.36, 95% CI 1.92–21.04) in monochorionic pregnancies and placental accreta (aOR 1.85, 95% CI 1.06–3.23) in dichorionic pregnancies. MCI was associated with an increased risk of preeclampsia (aOR 3.07, 95% CI 1.49–6.32), intertwin birthweight discordance ≥ 20% (aOR 2.40, 95% CI 1.08–5.60) and selective fetal growth restriction (aOR 2.46, 95% CI 1.08–5.60) in monochorionic pregnancies and small-for-gestational age neonates (aOR 1.97, 95% CI 1.24–3.14) in dichorionic pregnancies.

**Conclusions:**

VCI was associated with an increased risk of preterm delivery in twin pregnancies irrespective of chorionicity, whereas MCI was associated with an increased preeclampsia risk, significant intertwin birthweight discordance in monochorionic pregnancies and small-for-gestational age neonates in dichorionic pregnancies.

**Supplementary Information:**

The online version contains supplementary material available at 10.1186/s12884-023-05963-1.

## Background

The placenta is an essential temporary organ during pregnancy. As the link between the mother and the fetus, the placenta plays an important role in the transmission of nutrients and gases, endocrine synthesis, and immune barriers, is the primary regulatory element in the adaptation of the mother to pregnancy and maintains the intrauterine environment [1]. Hence, the development of placental structure and function is crucial for maternal and fetal health. Impairment of the placental structure or function may result in adverse short- and/or long-term outcomes, which include placental abnormalities (placental previa, accreta, abruption and abnormal morphogenesis) [2], placental-related diseases (preeclampsia (PE), preterm delivery (PTD), intrauterine growth restriction, fetal distress) [3–5] and increased risk for placenta-related chronic diseases in adulthood (diabetes, obesity and cardiovascular disease) [6].

Cord insertion abnormalities are common morphological abnormalities of the placenta where the umbilical cord does not insert into the center or the central neighborhood of the placental disk. The two main types of abnormal cord insertion are velamentous cord insertion (VCI) and marginal cord insertion (MCI), with a reported prevalence of 1.4% and 6.15%, respectively, in singleton pregnancies [7, 8]. Previous studies have revealed that VCI and MCI was associated with and increased risk of vasa previa, small-for-gestational age (SGA), PE, PTD, stillbirth and emergency cesarean delivery in singleton pregnancy [9–12]. Nevertheless, investigations on the impact of abnormal cord insertion in twin pregnancies have been primarily concerned with the associations between VCI and the occurrence of twin-to-twin transfusion syndrome (TTTS) and intertwin birthweight discordance (BWD) [13–15]. As abnormal cord insertion is more prevalent in twin pregnancies than in singleton pregnancies [12], it is essential to compare the influence of VCI and MCI on perinatal outcomes in twin pregnancies.

Based on a large-scale, single-center retrospective cohort of twin pregnancies in China, this study aims to estimate the associations of VCI and MCI with perinatal outcomes to provide evidence for obstetricians to make optimal decisions in clinical management.

## Methods

### Study population

This was a retrospective cohort study conducted in the Women and Children’s Hospital of Chongqing Medical University between January, 1 2017 and December 31, 2022. Twin pregnancies were identified by searching the Inpatient Electronic Medical Record (EMR) system, and all data were extracted and input into our database constructed by using Epidata 3.1. Twin pregnant women with any of the following conditions were excluded from the current study: delivery < 26^0/7^ weeks of gestation; congenital anomalies of one or both fetuses; preexisting chronic diseases, such as diabetes and hypertension and incomplete electronic medical records. Informed consent was obtained from all participants. The study was approved by the Ethics Committee of the Women and Children’s Hospital of Chongqing Medical University (No. 2022-011).

### Definition of abnormal cord insertion

Abnormal cord insertion was identified by abdominal ultrasound (captured from the admission diagnosis in EMR system) and confirmed after placental delivery (captured from the postpartum diagnosis in EMR system). VCI is characterized by the attachment of the umbilical cord to the amniotic membrane before reaching the placental disk, and the fetal blood vessels stretch along the membrane between the insertion site and the placenta [15]. MCI is characterized by an umbilical cord attachment site was < 2.0 cm to the nearest placental disk edge [15]. Twin pregnancies were grouped according to the cord insertion types. The VCI group included pregnancies complicated with velamentous insertion in one or both of the fetuses irrespective of the marginal cord insertion, and the MCI group included pregnancies complicated with marginal insertion in one or both of the fetuses. Twin pregnancies with all other umbilical cord insertion sites ≥ 2.0 cm from the nearest placental disk edge were categorized as the normal cord insertion (NCI) group.

### Study variables and outcomes

The following general data were collected: maternal prepregnancy age, height, weight, nulliparity, mode of conception (spontaneous conception or assisted reproductive technology (ART) use), chorionicity (monochorionic or dichorionic), hypertensive diseases of pregnancy (HDP), gestational diabetes mellitus (GDM), intrahepatic cholestasis of pregnancy (ICP) status, TTTS, selective fetal growth restriction (sFGR), gestational age (GA), premature rupture of membranes (PROM), placental previa, placental abruption, placental accreta, neonatal birthweight, fetal distress, fetal death and neonatal intensive care unit (NICU) admission.

Body mass index (BMI) was calculated as body weight/the square of height. GDM was diagnosed according to the International Association of the Diabetes and Pregnancy Study Groups criteria via a 75 g oral glucose tolerance test (OGTT) [16]. ICP was defined as elevated serum total bile acid level ≥ 10 µmol/L with pruritus [17].GA was determined by the larger fetus’s crown–rump length (before 14 weeks gestation) or head circumference (after 14 weeks of gestation) in the spontaneous conception cases, and by the timing of in vitro fertilization in the ART cases. Gestational weight gain (GWG) was calculated as the maternal weight prior to delivery minus the prepregnancy weight. Intertwin BWD was calculated as (larger twin birthweight-smaller twin birthweight)/larger twin birthweight × 100%, and intertwin BWD greater than 20% was consider as significant. Gestational hypertension (GHP) was defined as a systolic blood pressure ≥ 140 mmHg and/or diastolic blood pressure ≥ 90 mmHg after 20 weeks of gestation [18]. PE was defined as new onset of hypertension and proteinuria or significant end-organ dysfunction after 20 weeks of gestation [18]. PTD was defined as gestational age 34–37 weeks, 32–34 weeks and < 32 weeks. SGA was defined as a birthweight < 10th percentile corrected for GA and sex based on a Chinese twin-specific growth chart [19]. sFGR was defined as an intertwin BWD greater than 20%, with one of the neonates being SGA in monochorionic twin pregnancies.

### Statistical analysis

Data for continuous variables are presented as the mean (standard deviation) and those from categorical variables are presented as frequencies (percentages). Comparisons among the NCI, VCI and MCI groups were performed by one-way ANOVA for continuous variables and the chi-square test for categorical variables. The Bonferroni’s test was utilized as a post hoc analysis for one-way ANOVA.

We applied binary logistic regression models to examine the association between VCI and MCI and maternal outcomes. We used multinomial logit regression models to assess the association between VCI and MCI and PTD. Associations between VCI and MCI and neonatal outcomes were assessed by a generalized estimating equations (GEE) model to address the intertwin correlation. The effect estimates are reported as odds ratios (ORs) for binary logistic regression and GEE models and as relative risk ratios (RRRs) for multinomial logit regression models.

All statistical analyses were performed using Stata 15.0 (StataCorp, College Station, TX, USA).

## Results

**Fig. 1:**
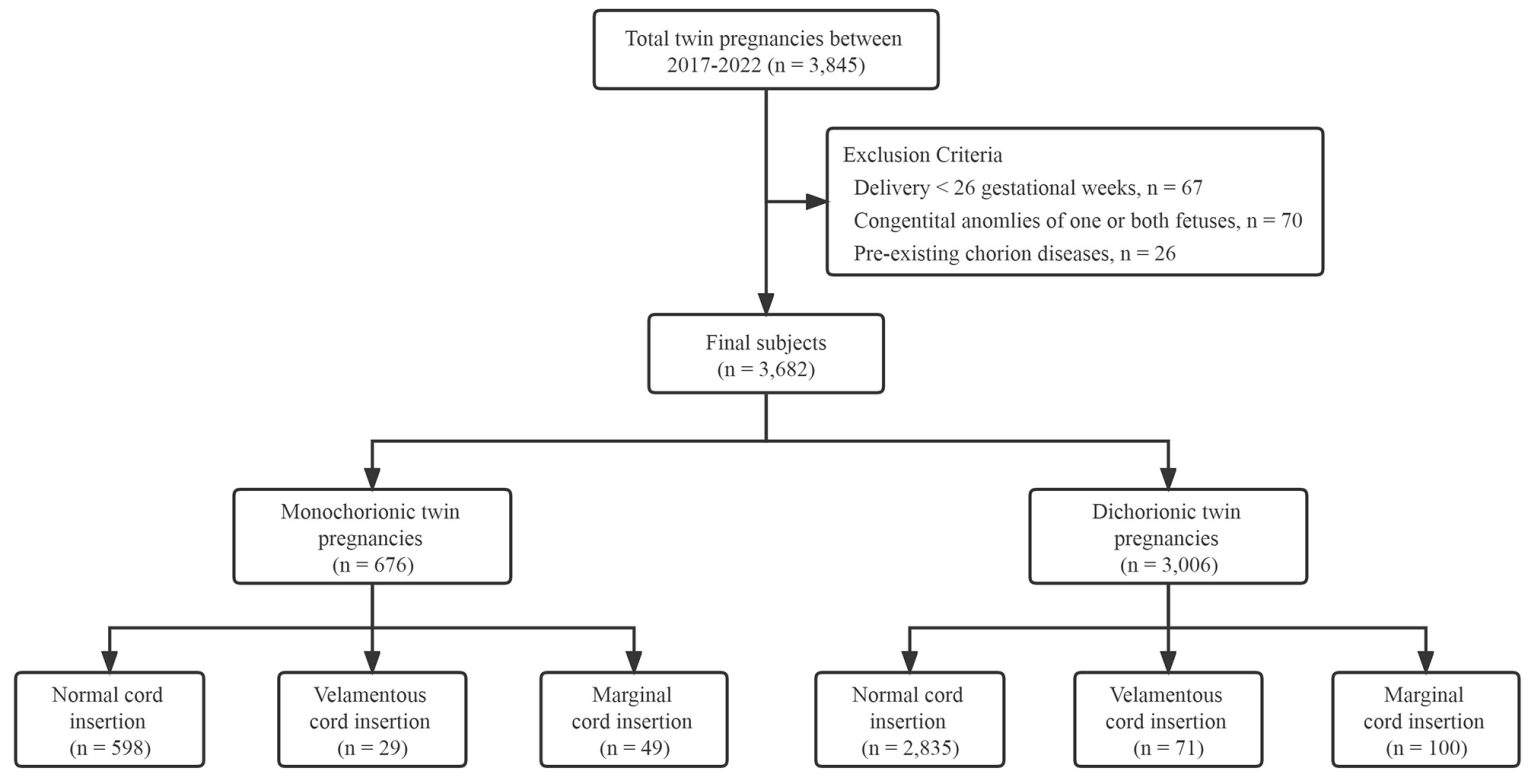
Flowchart showing selection of participants in final analysis

A total of 3682 twin pregnancies were included in the current study, including 100 (2.7%) with VCI and 149 (4.0%) with MCI (Fig. 1). Table 1 presents the comparisons of baseline characteristics, perinatal outcomes and neonatal outcomes among the groups. Compared to pregnancies with NCI, pregnancies with VCI or MCI accounted for significantly higher proportion of monochorionic twin pregnancies (29.0% vs. 17.4%, *p* = 0.003 and 32.9% vs. 17.4%, *p* < 0.001), spontaneous conception (47.0% vs. 28.0%, *p* < 0.001 and 40.3% vs. 28.0%, *p* = 0.001), PTD < 32 weeks (14.0% vs. 6.0%, *p* = 0.003 and 10.7% vs. 6.0%, *p* = 0.024), delivered at an earlier GA (34.89 ± 2.68 vs. 35.80 ± 2.12, *p* < 0.001 and 35.34 ± 2.58 vs. 35.80 ± 2.12, *p* = 0.033), lower birthweight (2217.07 ± 538.38 vs. 2392.31 ± 467.56, *p* < 0.001 and 2269.78 ± 515.72 vs. 2392.31 ± 467.56, *p* < 0.001) and a higher rate of NICU admission (49.0% vs. 28.9%, *p* < 0.001 and 45.6% vs. 28.9%, *p* < 0.001). Moreover, PTD was classified into two subcategories: spontaneous PTD and iatrogenic PTD. The majority cases of PTD were attributed to spontaneous PTD. Only in the VCI group, the rates of spontaneous PTD in the 32–34 week (14.0% vs. 6.1%, p = 0.004) and the < 32 week (12.0% vs. 4.6%, p = 0.003) were significantly higher than those in the NCI group (Supplementary Table 1).

**Table 1:** Comparison of baseline characteristics, perinatal outcomes and neonatal outcomes in twin pregnancies with or without abnormal cord insertion

Characteristics	NCI group (N = 3433)	VCI group (N = 100)	MCI group (N = 149)	*p* ^1^	*p* ^2^	*p* ^3^
Baseline characteristics						
Age at delivery, years	30.90 ± 3.86	30.68 ± 4.33	30.49 ± 3.68	1.000	0.562	1.000
Prepregnancy BMI, kg/m^2^	21.64 ± 2.88	21.35 ± 2.97	21.32 ± 2.21	0.953	0.574	1.000
Nulliparity, n(%)	2814 (82.0)	77 (77.0)	125 (83.9)	0.134	0.504	
Chorionicity, n(%)				**0.003**	**< 0.001**	0.517
Monochorionic	598 (17.4)	29 (29.0)	49 (32.9)			
Dichorionic	2835 (82.6)	71 (71.0)	100 (67.1)			
Mode of conception, n(%)				**< 0.001**	**0.001**	0.293
Spontaneous	960 (28.0)	47 (47.0)	60 (40.3)			
ART use	2473 (72.0)	53 (53.0)	89 (59.7)			
GWG, kg	16.74 ± 5.41	15.42 ± 6.23	16.87 ± 5.63	0.051	1.000	0.118
Perinatal outcomes						
GDM, n(%)	957/3324 (28.8)	27/96 (28.1)	29/146 (19.9)	0.910	**0.024**	0.161
GHP, n(%)	88 (2.3)	3 (3.0)	3 (2.0)	0.743	1.000	0.687
PE, n(%)	439 (12.8)	11 (11.0)	27 (18.1)	0.652	0.062	0.151
ICP, n(%)	533 (15.5)	18 (18.0)	21 (14.1)	0.576	0.648	0.477
GA at delivery, wks	35.80 ± 2.12	34.89 ± 2.68	35.34 ± 2.58	**< 0.001**	**0.033**	0.333
34–37 wks, n(%)	1469 (42.8)	36 (36.0)	57 (38.3)	0.184	0.310	0.790
32–34 wks, n(%)	283 (8.2)	18 (18.0)	17 (11.4)	**0.002**	0.173	0.192
< 32 wks, n(%)	206 (6.0)	14 (14.0)	16 (10.7)	**0.003**	**0.024**	0.552
PROM, n(%)	682 (19.9)	29 (29.0)	33 (22.2)	**0.031**	0.530	0.234
Placenta previa, n(%)	106 (3.1)	7 (7.0)	6 (4.0)	**0.039**	0.468	0.386
Placenta abruption, n(%)	52 (1.5)	0 (0.0)	4 (2.7)	0.403	0.293	0.151
Placenta accreta, n(%)	563 (16.4)	22 (22.0)	19 (12.8)	0.171	0.258	0.058
Postpartum hemorrhage, n(%)	157 (4.6)	5 (5.0)	8 (5.4)	0.806	0.687	1.000
Neonatal outcomes						
TTTS, n(%)	25 (0.7)	3 (3.0)	0 (0.0)	**0.043**	0.624	0.064
sFGR, n(%)	46 (1.3)	3 (3.0)	8 (5.4)	0.160	**0.002**	0.533
Intertwin BWD, %	10.52 ± 8.54	12.55 ± 9.25	11.60 ± 10.07	0.063	0.423	1.000
Intertwin BWD ≥ 20%, n(%)	444 (12.9)	18 (18.0)	26 (17.5)	0.173	0.135	1.000
Birthweight, g	2392.31 ± 467.56	2217.07 ± 538.38	2269.78 ± 515.72	**< 0.001**	**< 0.001**	0.676
SGA, n(%)	460/6866 (6.7)	18/200 (9.0)	38/298 (12.8)	0.251	**< 0.001**	0.194
Fetal distress, n(%)	218/6866 (3.2)	9/200 (4.5)	14/298 (4.7)	0.304	0.177	1.000
Fetal death, n(%)	66/6866 (1.0)	2/200 (1.0)	8/298 (2.7)	0.719	**0.011**	0.329
NICU admission, n(%)	1982/6866 (28.9)	98/200 (49.0)	136/298 (45.6)	**< 0.001**	**< 0.001**	0.465

ART, assisted reproductive technology; BMI, body mass index; BWD, birthweight discordance; GA, gestational age; GDM, gestational diabetes mellitus; GHP, gestational hypertension; GWG, gestational weight gain; ICP, intrahepatic cholestasis of pregnancy; MCI, marginal cord insertion; NCI, normal cord insertion; NICU, neonatal intensive care unit; PE, preeclampsia; PROM, premature rupture of membranes; sFGR, selective fetal growth restriction; SGA, small-for-gestational age; TTTS, twin-to-twin transfusion syndrome; VCI, velamentous cord insertion

^1^Post-hoc (Bonferroni test): comparison between NCI and VCI group

^2^Post-hoc (Bonferroni test): comparison between NCI and MCI group

^3^Post-hoc (Bonferroni test): comparison between VCI and MCI group

Compared to pregnancies with NCI, pregnancies with VCI had a higher proportion of PROM (29.0% vs. 19.9%, *p* = 0.031), placenta previa (7.0% vs. 3.1%, *p* = 0.039) and TTTS (3.0% vs. 0.7%, *p* = 0.043). Compared to pregnancies with NCI, pregnancies with MCI had a lower proportion of GDM (19.9% vs. 28.8%, *p* = 0.024), a higher proportion of sFGR (5.4% vs. 1.3%, *p* = 0.002), SGA (12.8% vs. 6.7%, *p* < 0.001) and fetal death (2.7% vs. 1.0%, *p* = 0.011).

There were no differences between twin pregnancies with VCI and those with MCI in terms of in baseline characteristics, perinatal outcomes and neonatal outcomes.

Table 2 displays the associations between pregnancies with VCI or MCI and perinatal, and neonatal outcomes. After adjusting for maternal age, prepregnancy BMI, nulliparity, chorionicity, mode of conception, HDP, GDM and ICP, twin pregnancies with VCI were associated with an increased risk of PTD at 32–34 weeks (aRRR 2.68, 95% CI 1.42–5.05, *p* = 0.002), PTD at < 32 weeks (aRRR 3.27, 95% CI 1.64–6.51, *p* = 0.001), PROM (aOR 1.72, 95% CI 1.09–2.73, *p* = 0.020) and NICU admission (aOR 2.35, 95% CI 1.60–3.46, *p* < 0.001). Twin pregnancies with VCI were also associated with an increased risk of placenta previa (aOR 2.54, 95% CI 1.14–5.67, *p* = 0.022) after adjusting for maternal age, prepregnancy BMI, nulliparity, chorionicity and mode of conception.

**Table 2:** Binary logistic regression analysis of association between different types of abnormal cord insertion and perinatal outcomes

Variables	VCI group		MCI group	
	aOR/aRRR (95% CI)^4^	*p*	aOR/aRRR (95% CI)^4^	*p*
Perinatal outcomes				
GDM, n(%)^1^	1.03 (0.65–1.64)	0.889	0.64 (0.41–0.97)	**0.038**
PE, n(%)^2^	0.92 (0.48–1.74)	0.795	1.56 (1.01–2.42)	**0.049**
PTD				
34–37 weeks, n(%)^3^	1.01 (0.61–1.70)	0.960	0.89 (0.61–1.31)	0.554
32–34 weeks, n(%)^3^	**2.68 (1.42–5.05)**	**0.002**	1.52 (0.87–2.65)	0.143
<32 weeks, n(%)^3^	**3.27 (1.64–6.51)**	**0.001**	1.78 (0.95–3.34)	0.071
PROM, n(%)^3^	**1.72 (1.09–2.73)**	**0.020**	1.20 (0.80–1.81)	0.383
Placenta previa, n(%)^1^	**2.54 (1.14–5.67)**	**0.022**	1.37 (0.59–3.19)	0.468
Placenta accreta, n(%)^1^	1.59 (0.97–2.60)	0.066	0.81 (0.49–1.33)	0.402
Neonatal outcomes				
TTTS, n(%)^1^	3.05 (0.85–11.01)	0.088	N/A	N/A
sFGR, n(%)^1^	1.34 (0.38–4.64)	0.649	**2.46 (1.08–5.60)**	**0.032**
Intertwin BWD ≥ 20%, n(%)^3^	1.37 (0.79–2.37)	0.265	1.47 (0.94–2.28)	0.088
SGA, n(%)^3^	1.25 (0.76–2.08)	0.380	**1.87 (1.30–2.70)**	**0.001**
Fetal death, n(%)^3^	0.94 (0.13–6.76)	0.949	2.18 (0.84–5.67)	0.108
NICU admission, n(%)^3^	**2.35 (1.60–3.46)**	**< 0.001**	**2.00 (1.46–2.75)**	**< 0.001**

BWD, birthweight discordance; GDM, gestational diabetes mellitus; MCI, marginal cord insertion; NICU, neonatal intensive care unit; N/A, not applicable; PE, preeclampsia; PROM, premature rupture of membranes; PTD, preterm delivery; sFGR, selective fetal growth restriction; SGA, small-for-gestational age; TTTS, twin-to-twin transfusion syndrome; VCI, velamentous cord insertion

^1^Adjusted for maternal age, prepregnancy BMI, nulliparity, chorionicity and mode of conception

^2^Adjusted for maternal age, prepregnancy BMI, nulliparity, chorionicity, mode of conception and gestational diabetes

^3^Adjusted for maternal age, prepregnancy BMI, nulliparity, chorionicity, mode of conception, hypertensive diseases of pregnancy, gestational diabetes and intrahepatic cholestasis of pregnancy

^4^Associations between abnormal cord insertion and preterm delivery subgroups were assessed by multinomial logist regression model, reported as RRR (95% CI). Associations between abnormal cord insertion and SGA, fetal death and NICU admission were assessed by generalized estimating equations model, reported as OR (95% CI). Associations between abnormal cord insertion and other outcomes were assessed by logistics model, reported as OR (95% CI)

After adjusting for maternal age, prepregnancy BMI, nulliparity, chorionicity, mode of conception, HDP, GDM and ICP, twin pregnancies with MCI were associated with an increased risk of SGA neonates (aOR 1.87, 95% CI 1.30–2.70, *p* = 0.001) and NICU admission (aOR 2.00, 95% CI 1.46–2.75, *p* < 0.001). Additionally, in the adjusted model, twin pregnancies with MCI were less likely to be complicated by GDM (aOR 0.64, 95% CI 0.41–0.97, *p* = 0.038) and more likely to be complicated by PE (aOR 1.56, 95% CI 1.01–2.42, *p* = 0.049) and sFGR (aOR 2.46, 95% CI 1.08–5.60, *p* = 0.001).

As shown in Table 3, monochorionic twin pregnancies with VCI were associated with an increased risk of PTD at 32–34 weeks (aRRR 2.94, 95% CI 1.03–8.39, *p* = 0.044), NICU admission (aOR 2.00, 95% CI 1.06–4.19, *p* = 0.035) and placenta previa (aOR 6.36, 95% CI 1.92–21.04, *p* = 0.002) after adjustment. Monochorionic twin pregnancies with MCI were associated with an increased risk of PE (aOR 3.07, 95% CI 1.49–6.32, *p* = 0.002), intertwin BWD ≥ 20% (aOR 2.40, 95% CI 1.18–4.86, *p* = 0.016) and NICU admission (aOR 2.66, 95% CI 1.50–4.72, *p* = 0.035). There was no association between abnormal cord insertion and TTTS in monochorionic twin pregnancies, and only pregnancies with MCI were associated with higher risk of sFGR (aOR 2.46, 95% CI 1.08–5.60, *p* = 0.001).

**Table 3:** Binary logistic regression analysis of association between abnormal cord insertion and perinatal outcomes in monochorionic twin pregnancies

Variables	VCI group		MCI group	
	aOR/aRRR (95% CI)^4^	*p*	aOR/aRRR (95% CI)^4^	*p*
Perinatal outcomes				
GDM, n(%)^1^	0.75 (0.27–2.08)	0.586	0.67 (0.31–1.44)	0.307
PE, n(%)^2^	1.38 (0.40–4.83)	0.610	**3.07 (1.49–6.32)**	**0.002**
PTD				
34–37 weeks, n(%)^3^	0.85 (0.31–2.34)	0.754	0.77 (0.36–1.67)	0.510
32–34 weeks, n(%)^3^	**2.94 (1.03–8.39)**	**0.044**	2.10 (0.88–4.99)	0.093
<32 weeks, n(%)^3^	2.31 (0.59–9.08)	0.231	2.20 (0.82–5.88)	0.116
PROM, n(%)^3^	2.15 (0.90–5.15)	0.085	1.28 (0.59–2.76)	0.532
Placenta previa, n(%)^1^	**6.36 (1.92–21.04)**	**0.002**	0.72 (0.10–5.52)	0.748
Placenta accreta, n(%)^1^	1.07 (0.36–3.18)	0.909	0.55 (0.19–1.57)	0.260
Neonatal outcomes				
TTTS, n(%)^1^	3.49 (0.95–12.88)	0.061	N/A	N/A
sFGR, n(%)^1^	1.34 (0.38–4.64)	0.649	**2.46 (1.08–5.60)**	**0.032**
Intertwin BWD ≥ 20%, n(%)^3^	1.29 (0.42–3.89)	0.657	2.40 (1.18–4.86)	**0.016**
SGA, n(%)^3^	0.53 (0.16–1.75)	0.295	1.65 (0.91-3.00)	0.100
Fetal death, n(%)^3^	N/A	N/A	3.31 (0.99–10.72)	0.056
NICU admission, n(%)^3^	**2.00 (1.06–4.19)**	**0.035**	**2.66 (1.50–4.72)**	**0.001**

BWD, birthweight discordance; GDM, gestational diabetes mellitus; MCI, marginal cord insertion; NICU, neonatal intensive care unit; N/A, not applicable; PE, preeclampsia; PROM, premature rupture of membranes; PTD, preterm delivery; sFGR, selective fetal growth restriction; SGA, small-for-gestational age; TTTS, twin-to-twin transfusion syndrome; VCI, velamentous cord insertion

^1^adjusted for maternal age, prepregnancy BMI, nulliparity and mode of conception. ^2^adjusted for maternal age, prepregnancy BMI, nulliparity, mode of conception and gestational diabetes

^3^adjusted for maternal age, prepregnancy BMI, nulliparity, mode of conception, hypertensive diseases of pregnancy, gestational diabetes and intrahepatic cholestasis of pregnancy

^4^Associations between abnormal cord insertion and preterm delivery subgroups were assessed by multinomial logist regression model, reported as RRR (95% CI). Associations between abnormal cord insertion and SGA, fetal death and NICU admission were assessed by generalized estimating equations model, reported as OR (95% CI). Associations between abnormal cord insertion and other outcomes were assessed by logistics model, reported as OR (95% CI)

As shown in Table 4, pregnancies with VCI were associated with an increased risk of PTD at 32–34 weeks (aRRR 2.55, 95% CI 1.19–5.46, *p* = 0.016), PTD at < 32 weeks (aRRR 3.67, 95% CI 1.67–8.06, *p* = 0.001), NICU admission (aOR 2.51, 95% CI 1.59–3.95, *p* < 0.001) and placenta accreta (aOR 1.85, 95% CI 1.06–3.23, *p* = 0.032) after adjustment. Dichorionic twin pregnancies with MCI were associated with an increased risk of SGA neonates (aOR 1.97, 95% CI 1.24–3.14, *p* = 0.004) and NICU admission (aOR 1.93, 95% CI 1.31–2.84, *p* = 0.001).

**Table 4:** Binary logistic regression analysis of association between abnormal cord insertion and perinatal outcomes in dichorionic twin pregnancies

Variables	VCI group		MCI group	
	aOR/aRRR (95% CI)^4^	*p*	aOR/aRRR (95% CI)^4^	*p*
Perinatal outcomes				
GDM, n(%)^1^	1.17 (0.69–1.99)	0.556	0.62 (0.37–1.04)	0.068
PE, n(%)^2^	0.86 (0.41–1.82)	0.694	1.12 (0.63–1.99)	0.712
PTD				
34–37 weeks, n(%)^3^	0.97 (0.53–1.77)	0.919	0.82 (0.52–1.29)	0.387
32–34 weeks, n(%)^3^	**2.55 (1.19–5.46)**	**0.016**	1.10 (0.51–2.38)	0.802
<32 weeks, n(%)^3^	3.67 (1.67–8.06)	**0.001**	1.50 (0.67–3.37)	0.327
PROM, n(%)^3^	1.58 (0.92–2.73)	0.099	1.17 (0.72–1.91)	0.526
Placenta previa, n(%)^1^	1.51 (0.46–4.93)	0.495	1.74 (0.69–4.40)	0.243
Placenta accreta, n(%)^1^	**1.85 (1.06–3.23)**	**0.032**	0.94 (0.53–1.65)	0.820
Neonatal outcomes				
Intertwin BWD ≥ 20%, n(%)^3^	1.46 (0.77–2.76)	0.244	1.14 (0.64–2.04)	0.658
SGA, n(%)^3^	1.59 (0.88–2.89)	0.124	**1.97 (1.24–3.14)**	**0.004**
Fetal death, n(%)^3^	N/A	N/A	0.86 (0.11–6.54)	0.887
NICU admission, n(%)^3^	**2.51 (1.59–3.95)**	**< 0.001**	**1.93 (1.31–2.84)**	**0.001**

BWD, birthweight discordance; GDM, gestational diabetes mellitus; MCI, marginal cord insertion; NICU, neonatal intensive care unit; N/A, not applicable; PE, preeclampsia; PROM, premature rupture of membranes; PTD, preterm delivery; SGA, small-for-gestational age; VCI, velamentous cord insertion

^1^adjusted for maternal age, prepregnancy BMI, nulliparity and mode of conception. ^2^adjusted for maternal age, prepregnancy BMI, nulliparity, mode of conception, and gestational diabetes

^3^adjusted for maternal age, prepregnancy BMI, nulliparity, mode of conception, hypertensive diseases of pregnancy, gestational diabetes and intrahepatic cholestasis of pregnancy

^4^Associations between abnormal cord insertion and preterm delivery subgroups were assessed by multinomial logist regression model, reported as RRR (95% CI). Associations between abnormal cord insertion and SGA, fetal death and NICU admission were assessed by generalized estimating equations model, reported as OR (95% CI). Associations between abnormal cord insertion and other outcomes were assessed by logistics model, reported as OR (95% CI)

## Discussion

On the basis of a retrospective analysis of twin pregnancies cohort from a single-center, we discovered that VCI were associated with an elevated risk of PTD in both monochorionic pregnancies and dichorionic pregnancies, and a higher risk of placenta previa and placenta accreta in monochorionic and dichorionic twin pregnancies, respectively. In addition, twin pregnancies with MCI were associated with an elevated risk of PE, significant intertwin BWD and sFGR in monochorionic pregnancies, and an elevated risk of SGA neonates in dichorionic pregnancies.

It has been consistently reported that VCI was a risk factor for intertwin BWD ≥ 20% or ≥ 25% in monochorionic twin pregnancies [15, 20–23], while was not associated with significant intertwin BWD in dichorionic twin pregnancies [15, 22]. Only Lee et al. reported that the incidence of significant intertwin BWD was not distinct in twin pregnancies with or without VCI [24]. Regarding the association between MCI and significant intertwin BWD, conflicting findings had been reported [15, 20, 21]. Only Cambiaso et al. demonstrated that MCI was associated with an increased risk of intertwin BWD ≥ 25% in monochorionic twin pregnancies [20]. In the present study, we discovered that MCI but not VCI was associated with intertwin BWD ≥ 20% in monochorionic twin pregnancies, which contradicted the aforementioned studies. The heterogeneity of the study population could provide an explanation for this. The low rate of VCI (4.3% in monochorionic twin pregnancies) in our population may have resulted in an insufficient number of pregnancies with VCI, which could have led to our study being underpowered to detect this association.

Similar to the above reason, no association was found between pregnancies with VCI and SGA neonates, but MCI was found to be associated with SGA neonates in dichorionic pregnancies in this study. There are several possible mechanisms to explain this finding. One is that abnormal cord insertion compromises fetal blood flow, resulting in decreased nutrient and oxygen supply. Another is that abnormal cord insertion can lead to placental insufficiency, which may impact the development of the placenta.

The association between abnormal cord insertion and the incidence of TTTS has been extensively studied in monochorionic twin pregnancies. In two studies, researchers noted that twin pregnancies complicated with TTTS had a significantly higher proportion of VCI than control twin pregnancies [25, 26]. However, recent studies have shown that VCI is not a risk factor for the development of TTTS [13, 15, 23, 24, 27]. In this study, we found that the incidence of TTTS was higher in the group with VCI than in the group with NCI. However, after adjusting for multiple variables, we did not find a correlation between VCI and the occurrence of TTTS. Surprisingly, none of the pregnancies with MCI were complicated by TTTS in this study, and to our knowledge, there have been very limited studies exploring the association between MCI and TTTS. Taken together, these findings suggest that there is a minimal correlation between abnormal cord insertion and the development of TTTS in monochorionic twins.

Limited studies have been conducted to elucidate the association between abnormal cord insertion and the onset of PE. Investigations based on singleton pregnancies have reported conflicting data on this association [10, 12, 28]. To our knowledge, this was the first study to report that pregnancies with MCI had a higher likelihood of developing PE in monochorionic twin pregnancies. This association can be explained by the fact that insufficient utero-placental blood perfusion is one of the pathogeneses of PE, and MCI can cause placental insufficiency, thus resulting in an increased risk of PE. Regarding the lack of association between VCI and the onset of PE, we speculated that it may be attributed to the twin pregnancies with VCI giving birth prior to PE onset, and these women were prone to delivery before 34 gestational weeks supporting this point.

We also found a relationship between VCI and placenta previa existed in monochorionic twin pregnancies and a relationship between VCI and placenta accreta existed in dichorionic twin pregnancies. Only Lee et al. reported that the prevalence of placenta accreta was higher in dichorionic twin pregnancies with VCI, but they did not adjust for covariates in their analysis [24]. However, we cannot explain the underlying connection between VCI and placenta previa or placenta accreta, which seemed to be a coincidental event.

The main strength of our study was that it is the first study to evaluate the impact of different types of abnormal cord insertion on the perinatal outcomes and neonatal outcomes in twin pregnancies stratified by chorionicity. In addition, a single-center design reduced the bias in reports of abnormal placental pathology.

The main limitation of the study was that over 70% of the twin pregnant women conceived with the aid of ART in our center, and the proportion of monochorionic twin pregnancies was far below that of dichorionic twin pregnancies. Another limitation was that the proportion of twin pregnancies with VCI and MCI in our study was lower than that in previous studies [22, 24]. The generalizability of the findings was limited due to the heterogeneity of the study population, but the findings still hold some reference value for subsequent investigations. Last, there was a lack of data on cervical length during pregnancy, which is an important risk factor for preterm delivery. This lack of data could potentially serve as a confounding factor impacting the association between abnormal cord insertion and preterm delivery.

## Conclusions

In conclusion, our study demonstrated that both velamentous cord insertion and marginal cord insertion have a negative impact on perinatal and neonatal outcomes in twin pregnancies, with a more pronounced effect in monochorionic twin pregnancies. Prospective studies are needed to explore prevention strategies and to improve the perinatal and neonatal outcomes of twin pregnancies with abnormal cord insertion.

### Electronic supplementary material

Below is the link to the electronic supplementary material.

Supplementary Material 1

## Data Availability

The data that support the findings of this study are available from the corresponding author, but restrictions apply to the availability of these data, which were used under license for the current study, and so are not publicly available. Data are however available from the authors upon reasonable request and with permission of corresponding author (Wanglan120@outlook.com).

## Abbreviations

ART: Assisted reproductive technology
BMI: Body mass index
BWD: Birthweight discordance
EMR: Electronic Medical Record
GA: Gestational age
GDM: Gestational diabetes mellitus
GEE: Generalized estimating equations
GHP: Gestational hypertension
GWG: Gestational weight gain
HDP: Hypertensive diseases of pregnancy
ICP: Intrahepatic cholestasis of pregnancy
MCI: Marginal cord insertion
NCI: Normal cord insertion
NICU: Neonatal intensive care unit
OGTT: Oral glucose tolerance test
OR: Odds ratio
PE: Preeclampsia
PROM: Premature rupture of membranes
PTD: Preterm delivery
RRR: Relative risk ratio
sFGR: Selective fetal growth restriction
SGA: Small-for-gestational age
TTTS: Twin-to-twin transfusion syndrome
VCI: Velamentous cord insertion
