# Supplementary material for: Associations between velamentous or marginal cord insertion and risk of adverse perinatal outcomes in twin pregnancies: a retrospective cohort study
Source: BMC Pregnancy Childbirth. 2023 Sep 8;23:648. doi: 10.1186/s12884-023-05963-1 (PMC10486129; doi:10.1186/s12884-023-05963-1)
Supplement: Supplementary file 1 — Supplementary Material 1 [file 12884_2023_5963_MOESM1_ESM.docx]

Supplementary Table 1. Comparison of different forms of preterm delivery in twin pregnancies with or without abnormal cord insertion

| Characteristics | NCI group  (N=3433) | VCI group  (N=100) | MCI group  (N=149) | *p*^1^ | *p*^2^ | *p*^3^ |
| --- | --- | --- | --- | --- | --- | --- |
| Spontaneous PTD, n(%) |  |  |  |  |  |  |
| 34-37 wks, n(%) | 1125 (32.8) | 24 (24.0) | 36 (24.2) | 0.066 | **0.032** | 1.000 |
| 32-34 wks, n(%) | 210 (6.1) | 14 (14.0) | 14 (9.4) | **0.004** | 0.117 | 0.308 |
| <32 wks, n(%) | 157 (4.6) | 12 (12.0) | 11 (7.4) | **0.003** | 0.113 | 0.266 |
| Iatrogenic PTD, n(%) |  |  |  |  |  |  |
| 34-37 wks, n(%) | 344 (10.0) | 12 (12.0) | 21 (14.1) | 0.613 | 0.126 | 0.705 |
| 32-34 wks, n(%) | 73 (2.1) | 4 (4.0) | 3 (2.0) | 0.279 | 1.000 | 0.443 |
| <32 wks, n(%) | 49 (1.4) | 2 (2.0) | 5 (3.4) | 0.655 | 0.072 | 0.705 |

PTD, preterm delivery.

^1^Comparison between NCI and VCI group.

^2^Comparison between NCI and MCI group.

^3^Comparison between VCI and MCI group.
